# Contributions of Artificial Intelligence to Decision Making in Nursing: A Scoping Review

**DOI:** 10.1111/nhs.70308

**Published:** 2026-02-18

**Authors:** Filipe Fernandes, Lucy Shinners, Mauro Mota, Paulo Santos, Luís Sá

**Affiliations:** ^1^ Institute of Health Sciences Catholic University of Portugal Porto Portugal; ^2^ Research Unit on Artificial Intelligence & Health, ESSVA‐IPSN/CESPU, Famalicão Vila Nova de Famalicão Portugal; ^3^ Faculty of Health Southern Cross University Gold Coast Australia; ^4^ Polytechnic University of Viseu, School of Health Viseu Portugal; ^5^ Higher School of Health of the Portuguese Red Cross (ESSCVP)—Lisbon. Health Sciences Research Unit: Nursing (UICISA: E) and Institute of Health Sciences, Center for Interdisciplinary Health Research (CIIS) Lisboa Portugal; ^6^ Portuguese Catholic University. Institute of Health Sciences, Center for Interdisciplinary Health Research (CIIS) Porto Portugal

**Keywords:** artificial intelligence, decision making, nursing

## Abstract

Recognizing the complexity of decision‐making is essential in nursing practice, where Artificial Intelligence (AI) can serve as a valuable tool to support nurses in the process of decision‐making. This scoping review aims to map and systematize evidence regarding AI's contributions to nursing decision‐making, following the Joanna Briggs Institute (JBI) methodological approach. Databases consulted: CINAHL Complete; MEDLINE Complete; Nursing & Allied Health Collection: Comprehensive; Cochrane Databases; MedicLatina; SciELO; Scopus; LILACS; JBI Database of Systematic Reviews and RCAAP. Thirteen studies in English, Portuguese, and Spanish were included. AI can support the nursing decision‐making process by improving diagnostic accuracy and workflows. However, interpretability remains a limiting factor that affects the adoption of AI. Although critical healthcare units represent the primary areas of application, meeting the ethical, legal, and technical requirements necessary for effective integration into practice continues to be a challenge. AI offers meaningful contributions to nursing decision‐making, particularly through explainable and clinically aligned systems. However, successful integration demands transparency, ethics, and usability, with further studies to ensure safe adoption.

## Key Points

- Artificial Intelligence can leverage decision‐making, enhancing nurses' skills.
- Nevertheless, the diverse array of risks posed by AI‐driven technologies needs comprehensive regulation.
- Regulating liability and ensuring equitable access to AI‐driven healthcare solutions are essential for building trust and inclusivity.

## 1 Introduction

Nursing profession has consistently addressed new challenges and thus the need to equip nurses with updated knowledge and enhanced skills based on the best available scientific evidence. Nurses now bear greater responsibility and autonomy in decision‐making, as reflected in professional practice regulations throughout the world (Brennan and Bakken 2015).

Historically, cognitive and social sciences explored how conscious and unconscious mechanisms influence behavior and decision‐making (Newen and Vogeley 2003). These early contributions helped establish the groundwork for understanding the neurological and psychological bases of decision‐making. More recent research has expanded this foundation by integrating neuroscience, behavioral science, and human factors engineering to better explain the complexity of clinical reasoning in nursing (Balconi 2020; van Gaal and Lamme 2012).

Thus, understanding the complexity of decision‐making is crucial for nursing and clinical practice. It allows nurses to identify points for improvement and reduce the possibility of error. This complex process calls for a harmonious combination of knowledge, experience, critical thinking, intuition, creativity, empathy, compassion, and the emotional as well as relational dimensions of care (Buchanan et al. 2020, 2021).

Contemporary studies show that many countries continue to invest in strengthening nurses' decision‐making skills to keep pace with evolving healthcare practice demands (Kelly et al. 2019; Lee et al. 2020). However, research also highlights the ongoing need to explore factors influencing decision‐making, including environmental complexity (often increases behavioral variability and demands more flexible, deliberative decision‐making strategies), cognitive load (that can lead the nurse to rely on heuristics rather than systematic, analytical thinking), and technological integration (e.g., information overload), that may impair decision quality (Shahsavarani et al. 2015).

Thus, despite the growing incorporation of AI in healthcare, it remains unclear how these tools have been studied and applied specifically in the nursing decision making process. The available evidence is fragmented, methodologically heterogeneous, and distributed across different practice contexts, making it difficult to systematically understand the impact of IA on the profession's decision‐making process. This gap justifies the need for a comprehensive review to map and critically organize existing knowledge.

### 1.1 The Review

Today it's undeniable that all caregivers and healthcare organizations must adopt systems and tools arising from technological advances to enhance their decision‐making and thus improve quality of care.

Artificial intelligence (AI) represents a transformative technology with the potential to enhance nursing care quality by supporting decisions related to diagnosis, risk assessment, and patient management, ultimately improving efficiency and safety while reducing costs (Douthit et al. 2020). According to Markets and Markets (2024), AI in the healthcare sector is expected to grow from 20.9 billion USD in 2024 to 148.4 billion USD by 2029 (Markets and Markets. 2024). This growth will inevitably impact nursing care, making it essential for nurses to understand these new technological tools and their implications in their daily practice. With the advent of artificial intelligence, nursing practice is experiencing significant advancements within contemporary healthcare. AI has the potential to revolutionize healthcare delivery, improve patient outcomes, and transform nurse's roles (Ronquillo et al. 2021). Nevertheless, regulation is crucial and needed. In November, the World Health Organization (WHO) issued guidelines on vital regulatory considerations for AI in healthcare. The document underscores the importance of safety, reinforcing the need for comprehensive legal and regulatory frameworks to safeguard privacy, security, efficacy, and collaboration while mitigating risks linked to AI's use of health data. It presents a global perspective on critical areas, including transparency, risk management, external data validation, commitment to data quality, and the challenges of navigating complex regulations such as the General Data Protection Regulation (GDPR) and the Health Insurance Portability and Accountability Act (HIPAA). Furthermore, it highlights the significance of fostering stakeholder collaboration (World Health Organization 2025).

AI tools are designed to mimic human thinking (Tang et al. 2023), helping the user to anticipate, predict, and facilitate decision‐making (Maalouf et al. 2018; McGrow 2019). AI‐powered clinical decision support systems provide healthcare professionals with valuable insights and evidence‐based recommendations, assisting them in making informed decisions about patient care (Van Bulck et al. 2023). Research has demonstrated AI's effectiveness in diagnoses and treatments by analyzing extensive datasets and identifying patterns, improving patient monitoring through predictive analytics (Buchanan et al. 2020). AI‐enabled technologies allow nurses to monitor vital signs continuously, detect early warning signs of clinical deterioration, and receive real‐time alerts, supporting timely interventions and reducing the risk of adverse events (Alazzam et al. 2022; McGrow 2019). It can also streamline care coordination by automating administrative tasks, prioritizing patient needs, and facilitating communication within healthcare teams (Stokes and Palmer 2020). This reduces nursing workload and enables more patient‐focused care, thus allowing nurses to dedicate more time to meaningful patient interactions (Barrera et al. 2020).

**FIGURE 1:**
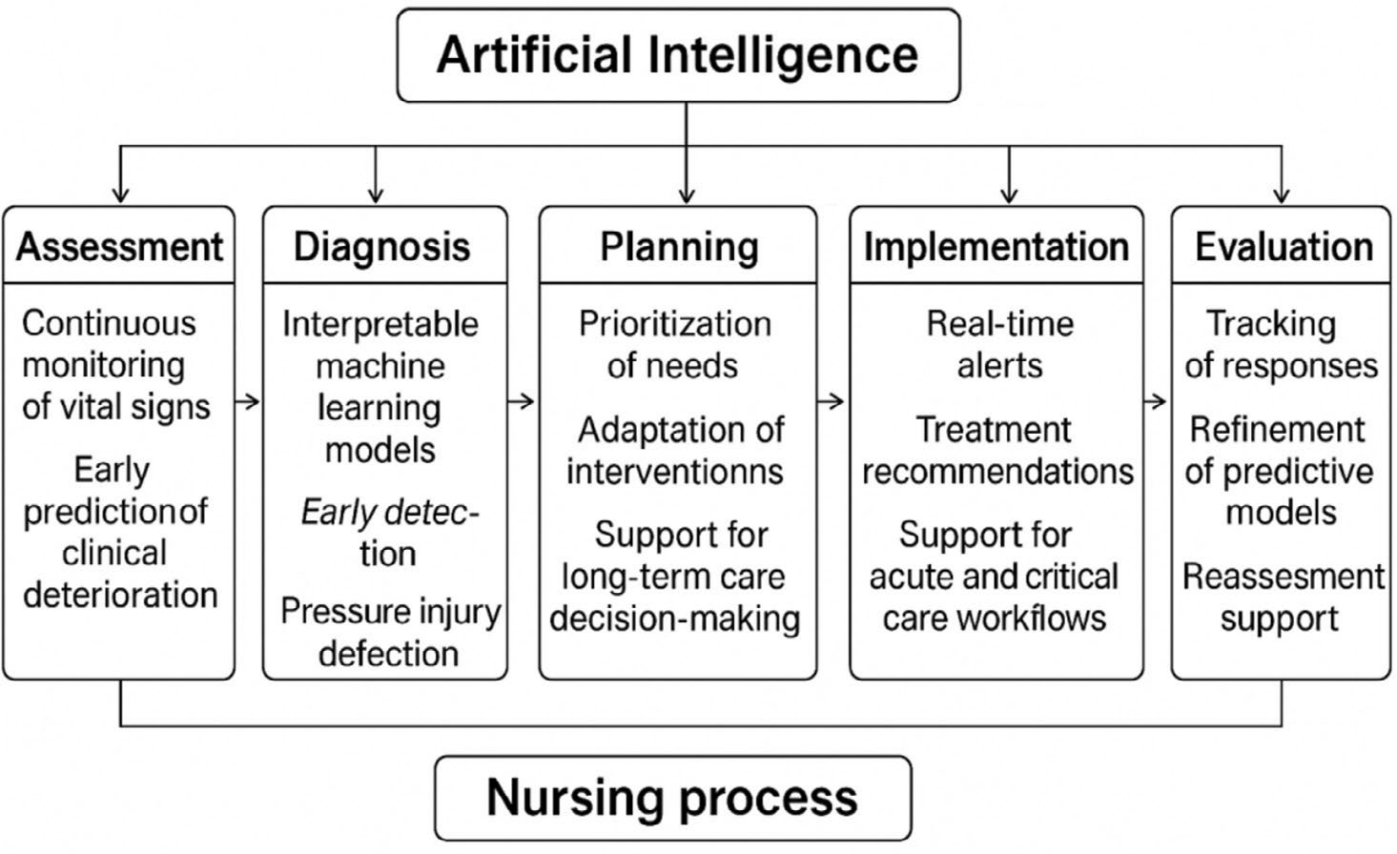
Application of AI across the stages of the nursing process.

Therefore, Clinical Decision Support Systems (CDSS) are essential in assisting healthcare professionals by providing data‐driven insights that facilitate rapid and informed decision‐making. These systems combine a diversity of physiological data, allowing the discovery of hidden patterns that might go unnoticed. By doing so, CDSS aids health professionals in making patient‐specific predictions, such as assessing in‐hospital mortality risk for intensive care unit (ICU) patients. However, traditional CDSS are frequently restricted to specific datasets and may need comprehensive integration of physiological knowledge. Recent advancements seek to incorporate expert knowledge into CDSS, increasing their ability to offer more reliable and insightful support in health decision‐making (Jalali et al. 2016). The concept of Explainable Artificial Intelligence (XAI) has emerged with the evolution of CDSS, as an essential framework for improving the transparency and interpretability of AI systems in healthcare. XAI methods aim to explain the decision‐making processes of AI models, making their outputs understandable to health professionals and patients. A process of critical importance in clinical settings, where trust and accountability are fundamental to practice. By enhancing the transparency of AI systems, XAI promotes greater confidence in AI‐assisted decisions, ensuring that healthcare providers can trust these tools for critical tasks such as diagnosis and treatment planning (Sadeghi et al. 2024). Specifically, in the nursing field, AI is intended to support and empower nurses, offer innovative ways to advance patient care and can contribute at each stage of the nursing process, enhancing decision‐making and thus enabling more confident choices (Figure 1).

Therefore, a more comprehensive understanding of how this tool influences clinical processes is still required, particularly in overcoming apprehension and distrust surrounding new AI technologies. Systematically mapping current evidence on AI's contributions to nursing decision‐making will help resolve existing concerns and provide greater clarity. By supporting evidence‐based practice and delivering real‐time data, AI empowers nurses to enhance overall care quality and effectively navigate the evolving healthcare landscape (Booth et al. 2021; Pepito et al. 2020; Randhawa and Jackson 2020). Reason why, it is of up most importance the development of studies in the field.

## 2 Aim

The primary aim of the scoping review is to map and systematize the published evidence regarding AI's contributions to nursing decision‐making. The secondary objectives are three, as follows: (i) Analyze the methodological designs used in studies investigating the application of AI to nursing decision‐making; (ii) Characterize the clinical, educational, and managerial scenarios in which AI has been used to support nursing decision‐making; and (iii) identify barriers, facilitators, and other factors that influence the adoption of AI in the nursing decision‐making process.

## 3 Methods

### 3.1 Reporting Method

This review was conducted following the JBI methodology for scoping reviews Peters et al. (2020) and the results were reported according to the guidelines of the Preferred Reporting Items for Systematic and Meta‐Analyses extension for Scoping Reviews (PRISMA‐ScR). This review was conducted in accordance with an a priori protocol, Fernandes et al. (2023) registered in the Open Science Framework (OSF) platform (osf.io/3zgcd).

A scoping review employs a systematic, iterative methodology to locate and synthesize both established and developing bodies of literature on a given subject. This scoping review was conducted between July and August 2024 in accordance with the JBI methodology (Peters et al. 2020). All studies published in Portuguese, English, and Spanish were considered for inclusion. Using a standardized framework, data were extracted, evidence levels assessed, study quality critically reviewed, and findings analyzed to provide a synthesis and overview of the literature. In addition, this review adhered to the Equator Network's PRISMA‐ScR reporting guidelines, ensuring transparency and consistency in reporting (Tricco et al. 2018).

The protocol was registered in October 2022, in the Open Science Framework (OSF) platform (https://osf.io/3zgcd/overview).

### 3.2 Search Strategy for Identifying Relevant Studies

The process followed a structured sequence of steps, namely the formulation of the review question; definition of eligibility criteria where participant, the concept under investigation, the context, and the sources of evidence were considered. Subsequently, the development of the search strategy was conducted in the following databases: CINAHL Complete; MEDLINE Complete; Nursing & Allied Health Collection: Comprehensive; Cochrane Central Register of Controlled Trials; Cochrane Database of Systematic Reviews; Cochrane Methodology Register; Library, Information Science & Technology Abstracts; MedicLatina (via EBSCO); SciELO; Scopus; LILACS; and JBI Database of Systematic Reviews databases. To find unpublished studies, an exhaustive search in the RCAAP database was fulfilled (The full search strategy is provided in Supporting Information: Appendix 1). This research was conducted in three stages.

First, an initial search of the MEDLINE and CINAHL databases (via EBSCO) was performed to identify critical descriptors and keywords commonly used with the MESH terms. In the second step, a search strategy was developed by blending keywords and indexed terms suited to each database. The third stage, bibliographic references of selected studies were reviewed to include additional relevant studies. All citations were managed using Mendeley Desktop (version 1.19.5), allowing duplicate studies to be removed. Thereafter, titles and abstracts were screened by two independent reviewers (B.M. and R.C.), with a third reviewer (J.J.) solving any discrepancies. Eligible documents were read in total, and exclusion options were documented. The search occurred between July and August 2024, with no geographical or time limitations.

### 3.3 Inclusion and Exclusion Criteria

The PCC [Population, Concept and Context] framework was used.

#### 3.3.1 Participants

This review considered all studies involving nurses working in the healthcare sector, regardless of age, gender, employment status, or work experience.

#### 3.3.2 Concept

This review considered all studies addressing the use of AI in decision‐making in nursing.

Decision‐making in nursing is defined as a structured cognitive process through which nurses analyze, interpret, and act upon complex clinical information to provide evidence‐based, patient‐centered care. Ronquillo et al. (2021) and Shahsavarani et al. (2015) highlight decision‐making as an integration of data, experience, and clinical guidelines to determine optimal patient outcomes. Douthit et al. (2020) describe it as a dynamic process where AI‐powered tools enhance nurses' ability to process real‐time information, improving precision and efficiency. Saban and Dubovi (2024) expand this definition by emphasizing the reduction of cognitive load through AI models that offer predictive insights and tailored recommendations, whereas Van Bulck et al. (2023) underscore its evolution with AI applications that enable proactive and context‐sensitive decisions. Decision‐making, therefore, is a critical and adaptive process in nursing that is increasingly supported by AI technologies to ensure high‐quality healthcare delivery.

#### 3.3.3 Context

This review considered studies related to hospital care where AI is used, regardless of the public or private nature of the institution. Studies addressing AI in nursing decision‐making, employing quantitative, qualitative, or mixed approaches, and published in English, Portuguese, or Spanish were considered eligible. All studies in other languages were excluded due to time and financial constraints for translations. Although currently there are online translators, translation through these AI‐powered tools may lead to misinterpretations and compromise the rigor of the review due to uneven translation quality. Regarding the exclusion criteria, all studies that did not include AI.

### 3.4 Data Collection

Two independent reviewers extracted data for the scoping review after confirming the relevance of selected publications. A researcher‐developed tool, detailed in the scoping review protocol (Fernandes et al. 2023), was used for data extraction, focusing on the population, concept, and context. The pilot screening process was conducted independently by both reviewers on 20 initial titles and abstracts to clarify the inclusion criteria.

## 4 Results

**FIGURE 2:**
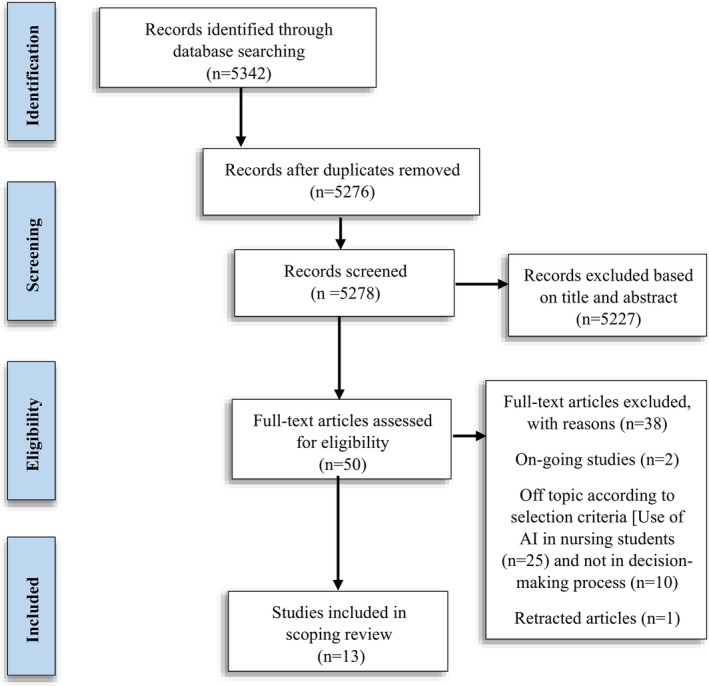
Flowchart depicting the article selection process.

The search strategy yielded 5342 studies, of which 5227 were excluded for not addressing the study topic (Figure 1), remaining 51 articles. Of these, 37 were excluded based on ongoing studies (2), of topic according to selection criteria—use of AI in nursing students (25), not implied decision‐making process (10) and retracted study (1). The objective of this review is to examine the methods used to study the contribution of AI to decision‐making in nursing and explore the context in which the contribution of AI to decision‐making in nursing has been studied. Articles that did not focus on these criteria were excluded from the review. A total of 13 studies were finally included in the review (Figure 2).

### 4.1 Overview of Included Studies

The studies included covered a period between 2006 and 2024. Most studies are from 2024 (*n* = 4), 2023 (*n* = 3), 2020 (*n* = 2), and 2022, 2021, 2016, 2007, and 2006 (*n* = 1). The studies took place mostly in the United States of America (USA) (*n* = 5) (Dweekat et al. 2023; Jalali et al. 2016; Kang et al. 2021; Kovach and Pollonini 2022; Popernack 2006) but also in Israel (*n* = 1) (Kang et al. 2021) and the Netherlands (*n* = 1) (Lukkien et al. 2024). Some studies are reviews (*n* = 6) (Bharadwaj et al. 2021; Mahmoudi and Moradi 2024; Martinez‐Ortigosa et al. 2023; Sadeghi et al. 2024; Stiglic et al. 2020; Yelne et al. 2023). There are two retrospective cohorts from 2020 and 2023 (Dweekat et al. 2023; Kang et al. 2021). Four occurred in an ICU (*n* = 3) (Jalali et al. 2016; Kang et al. 2021; Kovach and Pollonini 2022). The principal findings are summarized in Table 1, categorized according to study type (original research and review articles).

**TABLE 1:** Overview of selected articles.

Authors year/country	Sample (N)/setting and roles	AI modality/clinical task	Type of study/objectives	Outcomes	Main results	Key contributions to nursing decision
Primary empirical studies						
Kang et al. (2021) United States of America.	Training Dataset (eICU): 198, 167 patients (2014–2015, multiple US hospitals) Validation Dataset (MIMIC‐III): 21, 139 patients (2001–2012, Beth Israel Deaconess Medical Center). Critical care nurses.	Deep learning/prediction (ICU mortality).	Retrospective cohort with predictive modeling. Develop a deep learning model for ICU mortality prediction that is both clinically practical and interpretable. Validate the performance of the model using an external dataset (MIMIC‐III) to ensure generalizability across different ICU populations. Enhance the interpretability of the model by incorporating a two‐level attention mechanism to identify significant risk factors for mortality. Implement the model in a decision support system to assist healthcare professionals in making real‐time, data‐driven clinical decisions in ICU settings.	Model accuracy: AUC of 0.855 on external validation (MIMIC‐III). Interpretability: Key clinical variables and timesteps identified via two‐level attention mechanism. Performance: Outperformed traditional models in prediction accuracy. Generalizability: Validated across different ICU datasets with slightly lower external performance. Clinical Support: Integrated for real‐time ICU decision‐making. Improvements: Potential for further model optimization noted.	The proposed two‐level attention‐based LSTM model achieved high accuracy in predicting ICU mortality, with an AUC of 0.855 in external validation using the MIMIC III dataset. Logistic regression (LR) served as a baseline but underperformed due to overfitting, with a lower AUC (0.741) in external validation. Compared to traditional models, the LSTM model showed significantly better performance in mortality prediction. Including variables such as SOFA and eGFR in the model improved the AUC by about 5%. The two‐level attention mechanism provided enhanced interpretability, identifying influential time steps and critical variables related to mortality. Oxygen saturation and bicarbonate levels were negatively correlated with mortality, serving as protective factors, while bilirubin and white blood cell counts were positively correlated, indicating higher risk. The study highlighted the model's utility in ICU clinical decision‐making, emphasizing its interpretability for physicians.	Supports early prioritization via interpretable risk trajectories.
Popernack (2006) United States of America.	Total Respondents: 81 ICU nurses. Adult ICU: 31 nurses. Pediatric ICU: 24 nurses. Neonatal ICU: 26 nurses.	Computerized provider order entry (CPOE)/Medication safety/workflow.	Descriptive cross‐sectional study/ Assess the impact of the Computerized Provider Order Entry (CPOE) system on ICU nurses' workflow, communication, and patient care. Evaluate ICU nurses' perceptions of the CPOE system's effectiveness in improving patient safety and clinical efficiency. Identify the advantages and disadvantages of the CPOE system in daily nursing practice. Provide insights and lessons learned for guiding the implementation of similar systems in other healthcare institutions.	Improved patient safety: Reduced errors and improved legibility. Mixed workflow efficiency: Some found it efficient, others found it complicated. Decreased communication: Less verbal interaction with physicians. Advantages: Faster medication processing, easier access to information. Challenges: Learning curve, double charting, system integration issues.	Enhanced patient safety: Reduced transcription errors, improved legibility, and integrated medication alerts improved safety. Communication challenges: 71.2% of nurses reported a negative impact on nurse‐physician communication, with less direct interaction due to remote order access. Mixed workflow efficiency: Despite quicker access to critical information, 56.7% of nurses felt workflow efficiency decreased, often due to double charting and time spent on computers. Impact on hands‐on care: Some nurses felt they had less time for direct patient care as they spent more time managing electronic records. Need for physician training: Nurses observed that physicians relied on them for system navigation, highlighting the need for additional training. Recommendations for implementation: Emphasize physician training on CPOE functionalities. Maintain clear, verbal communication to ensure critical orders are understood and timely. Adapt the system to meet ICU‐specific needs and high‐acuity workflows.	Enhances medication safety; requires workflow redesign; reduced communication
Kovach and Pollonini (2022) Unietd States of America.	Interviews: *N* = 8. Survey: *N* = 55. Shadowing: *N* = 2. Focus groups: smaller set of participants (exact number not detailed)/ICU/Bedside nurses.	Machine learning (sensor‐based device)/pressure injury detection.	Mixed methods study qualitative data collection techniques (such as shadowing, interviews, and focus groups) with quantitative data (through surveys) Identify the key design elements of a device to detect and communicate hospital‐acquired pressure injuries (HAPIs) to ICU nurses. Determine how this design elements should function to help ICU nurses intervene appropriately. Ensure the device minimizes workflow disruptions for ICU nurses while providing actionable and timely information. Support the future development of technologies that reduce the incidence and severity of pressure injuries.	Nurse Preferences for Device Features: ICU nurses expressed the need for a device that provides real‐time summaries of patient skin conditions. The device should communicate the severity of skin issues in an easy‐to‐understand manner. Nurses preferred reminders that prompt them to act when needed, but these should not unnecessarily interrupt their workflow. There was a strong preference for using visual signals (colors and minimal alarms) to reduce the risk of alarm fatigue. The survey revealed six top‐rated needs for the device, including: At‐a‐glance information about the patient's skin condition. Real‐time data with minimal disruption to workflow. Information that is easy to understand and can trigger actions by nurses. The ability to identify the severity of the issue quickly. The chosen design integrated color‐coded displays with existing electronic medical records (EMR). A device designed around ICU nurses' needs will likely improve adoption and usability. Devices based on these findings could reduce hospital‐acquired pressure injuries (HAPIs). Visual indicators and minimizing alarm exhaustion are key for successful integration into ICU workflows.	Nurses' preferences for device design: ICU nurses prefer a device that provides real‐time, at‐a‐glance summaries of skin condition severity, with minimal workflow disruption and easy‐to‐understand visual cues. Action‐triggering and reminders: The device should remind nurses to take necessary action without contributing to alarm fatigue; using visual signals (e.g., color‐coded indicators) instead of sounds was emphasized. Integration with EMR: Ideal designs include integration with EMR, showing a color‐coded body map for monitoring skin conditions and alerting staff with options to snooze, dismiss, or request assistance. User‐centred design process: The study highlighted the importance of involving nurses in the design process to ensure that devices align with their workflow, which may encourage higher adoption and use in clinical settings. Top needs identified: Critical features include real‐time updates, ease of use, minimal disruption, and capability for team support in ICU settings, addressing issues like alarm fatigue and workload management.	Supports timely detection through interpretable visual alerts
Jalali et al. (2016) United States of America.	*N* = 4000 adult ICU patients. Data source: PhysioNet database/ICU/Critical care nurses.	Neural network CDSS/prediction (mortality).	Clinical prediction model development study. Develop a clinical decision support system (CDSS) to predict in‐hospital mortality of ICU patients. Improve upon existing ICU scoring systems (e.g., SOFA, SAPS‐III) by creating a patient‐specific, real‐time prediction model. Integrate expert physiological knowledge into the algorithm for better clinical relevance and model interpretability. Provide more accurate and reliable decision support for healthcare professionals in ICU settings.	The new CDSS achieved a 42% F‐score and outperformed SOFA and SAPS‐III scoring systems by 55% and 45% respectively. The model effectively integrated expert physiological knowledge, improving prediction accuracy and clinical relevance. The CDSS provides more accurate, patient‐specific predictions compared to existing systems. Incorporating physiological insights into machine learning models improves their clinical applicability and interpretability.	New CDSS: A machine learning‐based CDSS was developed to predict ICU outcomes based on the first 48 h of physiological data. Superior prediction accuracy: The CDSS achieved a 42% F‐score, outperforming traditional scoring systems SOFA (27%) and SAPS‐III (29%) by approximately 45%–55%. Organ‐based feature grouping: Data was organized by organ system (e.g., heart, lung, kidney), enhancing the interpretability and relevance of the model for clinical use. Feature extraction and ranking: Statistical and demographic features, including out‐of‐range indices and alarm counts, were extracted and ranked to predict outcomes. Neural network model: A tailored neural network prioritized cardiac and neural indicators for ICU outcome predictions, demonstrating a model that integrates physiological expertise.	Supports diagnostic reasoning with organ‐level interpretability.
Dweekat et al. (2023) United States of America.	*N* = 15 889 patients, admitted to Christiana Care Hospital in Delaware, Inpatient acute care/Hospital nurses.	Machine learning/Risk and timing prediction.	Retrospective cohort study. Develop machine learning models to predict the occurrence of Hospital‐Acquired Pressure Injuries (HAPI) in hospitalized patients. Enhance prediction accuracy of HAPI using advanced techniques like Cost‐Sensitive Support Vector Machine (SVM), Genetic Algorithm (GA), and Grid Search (GS). Predict the timing of HAPI development to identify when patients are most likely to develop HAPI. Improve clinical decision‐making by providing early warnings, enabling healthcare professionals to implement preventive measures and reduce HAPI incidence.	Machine learning models accurately predicted the occurrence of Hospital‐Acquired Pressure Injuries (HAPI). The cost‐sensitive SVM optimized with Genetic Algorithm (GA) and Grid Search (GS) showed strong predictive performance. Models successfully predicted the timing of HAPI development, providing early warnings. High accuracy was demonstrated using metrics such as AUC, G‐mean, and sensitivity.	Dual‐phase machine learning model: Developed a two‐phase ML system to predict if and when HAPI occurs. Phase 1—identifying At‐risk patients: Utilized a GA with a Cost‐Sensitive Support Vector Machine (CS‐SVM) to handle the highly imbalanced dataset. This model achieved an AUC of 75.79 and a sensitivity of 74.29%, outperforming traditional models. Phase 2—predicting timing of HAPI: Employed Grid Search with SVM to predict the time of HAPI occurrence, classifying high‐risk patients who might develop HAPI within 7 days. The optimized SVM model achieved an AUC of 75.06. Top risk factors identified: Features like the Glasgow Coma Scale, feeding tube presence, number of surgeries, and Braden Scale subfactors were key predictors across both phases. Clinical utility: By predicting the timing of HAPI, the model enables healthcare teams to prioritize high‐risk patients, allocate resources effectively, and reduce patient harm and hospital costs.	Supports prevention and prioritization/enables anticipatory planning and resource allocation.
Lukkien et al. (2024) Netherlands	*N* = 24 participants/Long‐term care (LTC)/LTC nurses.	CDSS (rule‐based plus ML‐informed)/Care planning/monitoring.	Qualitative exploratory study Explore prerequisites for responsible AI‐assisted decision‐making in nursing practice, focusing on the factors necessary for its responsible use. Investigate the opportunities and risks of AI‐assisted decision‐making in long‐term care, identifying both benefits and challenges. Provide recommendations for responsible AI innovation in long‐term care settings, offering strategies for the design, implementation, and use of AI technologies.	AI‐based decision support systems (CDSS) offer potential to: Assist with early identification of care needs. Guide the development of personalized care strategies. Enhance shared decision‐making between caregivers and clients. Reduce caregiver workload by automating routine tasks, freeing time for empathetic care. Main risks: Overreliance on AI, reducing caregiver autonomy and privacy, bias, and depersonalization concerns AI can improve care quality but requires a balanced, collaborative approach to mitigate risks.	Opportunities in early identification and care strategies: CDSS help caregivers identify early care needs, guide care strategies, and improve shared decision‐making. Mixed reactions to AI in care: Professionals recognize potential benefits (like workload reduction) and risks (over‐reliance and depersonalization of care) in AI‐assisted decision‐making. Privacy and ethical concerns: There are significant concerns around privacy, autonomy, and biases in data, particularly regarding AI's impact on older adults' dignity and self‐determination. Seven prerequisites for responsible Use: Regular review of data collection to ensure relevance and minimize risks. Balancing AI's proactive nature with human judgment to avoid automation of decisions. Incremental deployment to build trust and align with user experience. Customization for various user roles ensures AI fits clients' and caregivers' needs. Measures to counteract biases and provide context‐aware insights. Human‐centered learning loops involving caregivers in AI training and improvements. Routinization of AI use in care, integrating AI insights naturally into decision‐making.	Highlights safe‐use prerequisites and ethical considerations.
Saban and Dubovi (2024) Israel	*N* = 68 participants (30 registered. Nurses and 38 nursing students)/Education setting.	LLM (ChatGPT)/Triage/decision. Comparison.	Cross‐sectional study/ Explore the potential of ChatGPT as a clinical support tool for nurses. Assess whether ChatGPT can demonstrate clinical decision‐making equivalent to that of expert nurses and novice nursing students. Compare ChatGPT's responses to clinical vignettes with those of expert and novice nurses. Evaluate ChatGPT's performance in terms of assessment of presenting signs and symptoms, differential diagnosis. Recommendations for diagnostic testing and revaluation based on new patient information.	ChatGPT showed indecisiveness in initial clinical assessments. It tended to over‐triage, suggesting unnecessary tests. ChatGPT's revaluations were often inaccurate or inappropriate when new information was introduced. Faster responses than novices and experts but with significantly more words. ChatGPT is not yet reliable for clinical decision‐making in complex, urgent scenarios. There are significant risks in using ChatGPT for clinical tasks without further improvement. It shows potential as a support tool but requires further training and optimization before safe clinical use.	AI versus human performance: ChatGPT was tested against expert nurses and students. It showed logical responses but was often indecisive and prone to over‐triage. Challenges in adaptation: ChatGPT sometimes struggled to adjust decisions when new clinical information was provided. Efficiency and wordiness: ChatGPT responded much faster than humans, but its answers were significantly longer. Educational potential: ChatGPT could support nursing education by offering structured reasoning, but it requires further refinement for reliable clinical use.	LLM shows potential for education; unsafe for autonomous decisions.
Reviews						
Sadeghi et al. (2024) NA.	113 studies were included. Multi‐setting.	Explainable AI (XAI)/Interpretability and transparency.	Systematic review Provide an exhaustive exploration of XAI applications in healthcare. Analyze relevant experimental outcomes related to XAI in the healthcare context. Foster a holistic understanding of XAI's role and it's potential in this critical domain.	XAI Methods: Feature‐oriented, global, concept, surrogate, pixel‐based, human‐centric. Challenges: High‐stakes decision‐making requires explainability. Risks from “black‐box” AI models. Need for trustworthy, real‐time decision‐making. Safety‐critical importance: Trust and interpretability are essential for ethical AI use. Experimental insights: Successful use in Parkinson's, sepsis, cancer predictions. XAI helps explain complex medical predictions. Role in decision‐making: Makes AI decisions understandable and supports clinical accuracy. Future research: Focus on transparency, hybrid models, and healthcare‐specific tools.	XAI method categories: Six main types tailored for healthcare: feature‐oriented, global, concept models, surrogate models, local pixel‐based, and human‐centric methods. Healthcare impact: XAI applications improve diagnosis and understanding of Parkinson's, Alzheimer's, lung cancer, and COVID‐19 through techniques like SHAP and LIME. Challenge of complexity versus Transparency: High‐performing AI models often lack transparency, highlighting the need for interpretable models in clinical settings. Ethics and trust: XAI enhances accountability and patient trust by addressing model biases and upholding ethical standards. Future research: Emphasis on developing self‐explanatory models for broader, reliable AI integration in healthcare.	Improves justification of AI‐assisted decisions.
Bharadwaj et al. (2021) India.	NA.	ML interpretability/Monitoring; predictive analytics.	Review Provide an overview of prominent machine learning (ML) algorithms and their applications in healthcare IoT. Review important applications of ML algorithms in diagnosing and prognosing common diseases. Investigate the role of ML in forecasting disease stages and controlling epidemics with IoT. Identify assistive systems for improving the quality of life for physically challenged, mentally disabled, and elderly individuals. Explore emerging ML‐IoT technologies for making healthcare systems more accessible and efficient.	Integration of machine learning with IoT can improve patient monitoring, diagnostics, and treatment personalization. Machine learning algorithms (e.g., neural networks, support vector machines) show success in healthcare applications like disease prediction and patient monitoring. Challenges include data privacy, model interpretability, and integration with existing healthcare systems. Explainable AI (XAI) is crucial for clinicians to trust and use AI‐driven tools effectively in healthcare. IoT and machine learning have the potential to transform healthcare, but further research is needed to address technical and ethical challenges for broader adoption.	Enhanced diagnostic capabilities: ML integrated with IoT in healthcare has proven highly effective in diagnosing cardiovascular and neurological disorders, diabetes, and lung cancer with improved accuracy and efficiency. Predictive power for prognosis and spread control: ML algorithms, especially with IoT, provide predictive capabilities for future disease stages and control of epidemic spread, particularly relevant for infectious diseases. Assistive systems for vulnerable populations: IoT‐ML applications have been identified to support physically challenged, mentally disabled, and elderly individuals, enhancing their quality of life through continuous monitoring and assistance. Futuristic health monitoring systems: Emerging technologies promise accessible, efficient health monitoring, integrating real‐time data analysis and proactive healthcare delivery, especially for chronic diseases like diabetes and kidney disease. Challenges and constraints: Despite advancements, implementing ML‐IoT applications faces challenges in data security, interoperability, and privacy, which must be addressed to realize their full potential in healthcare settings.	Supports proactive reasoning and risk anticipation.
Stiglic et al. (2020) Slovenia.	Multi‐setting.	ML interpretability/Interpretability framework.	Review Provide an overview of interpretability approaches ML models in healthcare. Categorize interpretability methods into model‐specific versus model‐agnostic and local versus global interpretability. Offer practical examples of how interpretability techniques are applied in healthcare. Highlight the importance of interpretability for ML adoption in clinical decision‐making to help healthcare professionals trust and understand ML predictions. Discuss the challenges and future directions in developing interpretable ML models, including ethical concerns and the need for responsible AI in healthcare.	Interpretability methods in ML can be classified into model‐specific and model‐agnostic approaches and further divided into local and global interpretability. Model‐agnostic techniques provide more flexibility as they can be applied to any ML model, whereas model‐specific methods are tied to specific types of models like decision trees or linear models. Local interpretability focuses on understanding individual predictions, while global interpretability offers insights into the overall behavior of a model. Examples of successful application of interpretability in healthcare include predicting patient outcomes, optimizing treatments, and improving disease screening. Challenges remain in developing interpretable ML models that are complex enough for accurate predictions yet transparent enough for healthcare professionals to trust and use in decision‐making. Interpretability is essential for the successful adoption of ML models in healthcare, as healthcare professionals need to understand the reasoning behind predictions. Ethical concerns such as biases in ML models and the need for transparency are critical and must be addressed for ML to be responsibly used in clinical settings. Future research should focus on improving interpretability techniques, particularly in complex models like deep learning, to ensure that ML models are both accurate and explainable in healthcare applications. The integration of interdisciplinary teams including healthcare professionals, AI developers, and policymakers is crucial for advancing responsible and interpretable AI in healthcare.	Importance of interpretability: Interpretable ML models are critical in healthcare due to their impact on patient care and decision‐making. Types of approaches: Model‐specific versus model‐agnostic: Specific to certain models or universally applicable. Local versus global interpretability: Focus on individual predictions or overall model understanding. Applications: Used across healthcare fields to assist with diagnoses, treatment planning, and disease assessment. Key challenges: Issues like computational complexity, scalability, and ensuring fairness remain obstacles. Future research: There is a strong induce for more interpretable, trustworthy ML models to support safe and effective healthcare decisions.	Supports understanding and trust in AI recommendations.
Yelne et al. (2023) India.	Multiple clinical settings.	General AI/Decision support; education.	Review Examine AI's impact on healthcare and nursing science, focusing on its potential in personalized care, diagnostic accuracy, predictive analytics, and telemedicine. Address challenges related to AI integration, including data privacy, algorithm biases, and the need for transparency. Highlight the importance of interdisciplinary collaboration between healthcare professionals, AI developers, and policymakers to optimize AI's role. Provide actionable recommendations for better AI integration into clinical practice and healthcare management, ensuring ethical standards and patient‐centred care.	AI has significantly improved personalized care, diagnostic accuracy, predictive analytics, and telemedicine. Early detection and intervention using AI enhance diagnosis, treatment precision, and patient safety. AI streamlines administrative tasks, clinical decision support, and healthcare operations, improving efficiency. AI should complement healthcare providers, not replace them, while maintaining the human touch in care. Ethical concerns like transparency, fairness, and data privacy require continuous attention. Successful AI adoption depends on collaboration between healthcare professionals, AI developers, and policymakers. AI holds promise for advancements in diagnostics and predictive analytics, but ongoing research and ethical considerations are essential.	Enhanced diagnostic accuracy: AI technologies improve diagnostic precision in medical imaging and early disease detection, benefiting areas like oncology and critical illness. Clinical decision support: AI‐powered tools assist in real‐time decision‐making for treatment options, reducing errors and supporting healthcare professionals. Personalized treatment: AI enables customized treatment plans by analyzing patient data, enhancing treatment effectiveness and minimizing adverse effects. Efficiency in patient monitoring: AI‐driven remote patient monitoring and fall prediction systems help manage patient care efficiently, especially for chronic conditions. And privacy challenges: The integration of AI raises concerns about data privacy, algorithmic bias, and the need for transparency and accountability in healthcare settings. Interdisciplinary collaboration needed: Successful AI adoption in healthcare relies on Collaboration across fields, from engineering to ethics, to ensure that AI benefits patient care ethically and effectively.	Improves accuracy; ethical concerns.
Mahmoudi and Moradi (2024) Iran.	63 studies/Acute and community care/Clinical nurses.	General AI/Monitoring; triage; remote care.	Review Investigate the application of Artificial Intelligence (AI) in nursing care. Explore how AI can reduce diagnostic errors. Assess the impact of AI on improving emergency response times. Evaluate how AI enhances the quality of patient care and psychological support. Analyze AI's role in enabling remote care for elderly patients through smart technology. Examine the use of AI in electronic health records. Explore AI's contribution to healthcare cost analysis. Assess the implementation of AI in smart hospitals and technologies	Reduction of diagnostic errors through AI integration. Improved patient outcomes with timely interventions based on AI‐generated early warnings. Enhanced emergency response times via quicker identification of critical health indicators. Effective use of AI in remote patient monitoring, especially for elderly care. Reduction of nurses' workload through AI‐bots and robots performing routine tasks. Identification of ethical challenges, including over‐reliance on AI and data privacy concerns.	Improved diagnosis and prediction: AI assistance in accurate disease diagnosis, medical image analysis, and early detection of complications, reducing human error in healthcare. Enhanced resource management: AI systems help optimize healthcare resources by automating scheduling, reducing paperwork, and improving workflow efficiency. Patient monitoring and safety: AI‐enabled monitoring devices assist nurses by continuously observing patients, predicting issues, and reducing risks such as falls, especially for elderly patients. Support for personalized care: AI‐driven systems enable designed care based on individual patient needs, including nutrition and medication recommendations. Education and training: AI enhances nursing education through virtual simulations, supporting the development of clinical skills and decision‐making. Ethical and privacy concerns: Challenges include maintaining patient privacy, addressing algorithm biases, and balancing technology use with human compassion in patient care.	Supports emergency decision‐making and workload reduction.
Martinez‐Ortigosa et al. (2023) Spain.	21 studies/nurses.	General AI/Decision support.	Systematic review Synthesize the available evidence on the applicability of artificial intelligence (AI) in nursing care. Explore the use of AI‐based systems in early disease detection and clinical decision‐making. Investigate how AI can support patient monitoring and optimize workflows in nursing practice. Assess the role of AI in improving nursing training and education. Evaluate the potential of AI to enhance care delivery, increase efficiency, and ensure high‐quality patient care. Address concerns related to AI replacing human interactions and functions in nursing	AI improves nursing practice through better early disease detection, clinical decision‐making, patient monitoring, and workflow efficiency. AI systems increase diagnostic accuracy and reduce administrative tasks, allowing nurses to focus more on direct patient care. AI tools, such as chatbots and image recognition systems, are effective in areas like wound care and continuous patient monitoring. AI‐based educational tools enhance nursing students' clinical decision‐making skills. The study emphasizes that AI should support, not replace, nursing roles, preserving the human element in care. Ethical concerns, including data privacy and the potential reduction in human interaction, need to be addressed for responsible AI implementation.	Enhanced diagnosis and decision‐making: AI supports early disease detection and improves accuracy in clinical decisions. Efficient patient monitoring: AI tools reduce provider workload through remote monitoring and support systems, especially in ICUs. Improved nursing education: AI simulations and chatbots help train nursing students, enhancing confidence and clinical skills. Positive impact with ethical cautions: AI improvements care quality and efficiency but raises concerns about data privacy and human‐centered design.	Improves workflow, decision‐making.

The main results are presented in alignment with the objectives of each study.

- The role of explainable AI in enhancing nursing decision‐making.

Interpretability and Transparency: Explainable AI (XAI) is crucial in healthcare since it allows health professionals to understand and trust the predictions and recommendations made by AI systems, especially in critical care settings, where decisions significantly impact patient outcomes (Kang et al. 2021; Stiglic et al. 2020).

Improving Trust and Accountability: XAI allows health professionals to understand the rationale behind AI recommendations, thus building trust and supporting ethical decision‐making in healthcare contexts like ICU and LTC (Kang et al. 2021; Lukkien et al. 2024).

Improving Clinical Adoption: XAI's role in making AI models interpretable can facilitate broader adoption by health professionals, enabling them to rely on AI tools more confidently in various clinical settings (Stiglic et al. 2020).

Educational Potential: XAI has shown potential as a learning tool for novice nurses, helping them understand clinical decision‐making processes by following structured, transparent reasoning (Martinez‐Ortigosa et al. 2023).

Enhanced Clinical Decision Support: AI's role in providing interpretable and transparent diagnostic insights improves its applicability in nursing by aiding in more informed and precise decision‐making (Mahmoudi and Moradi 2024).

B Methods Used to Study AI's Contribution to Nursing Decision‐Making

Machine Learning Models: Studies have employed diverse machine learning models, such as genetic algorithms combined with cost‐sensitive support vector machines (SVMs) and grid search optimization, to predict clinical events (e.g., hospital‐acquired pressure injuries) and guide nursing decisions (Dweekat et al. 2023; Martinez‐Ortigosa et al. 2023).

Clinical Decision Support System (CDSS): Evaluation of CDSS, including ICU‐specific models, highlights how advanced neural network models integrated with patient data can provide predictive insights, with specific testing on mortality prediction (Kang et al. 2021).

Quantitative, Qualitative and Mixed Methods Analyses: Studies employ mixed methods, including qualitative interviews with nursing professionals in LTC and quantitative comparative studies in ICUs to evaluate AI‐assisted decision‐making (Kovach and Pollonini 2022; Lukkien et al. 2024; Saban and Dubovi 2024).

Comparative Studies: ChatGPT's performance was compared with human practitioners in emergency care settings, highlighting AI's speed, verbosity, and indecisiveness in clinical assessments (Saban and Dubovi 2024).

C Contexts in which AI's contribution to nursing decision‐making has been studied.

Critical Care and ICU Settings: Much of the research focuses on ICU environments where AI tools assist with mortality prediction, patient monitoring, and timely alerts for critical interventions (Kovach and Pollonini 2022; Popernack 2006), supporting high‐stakes decision‐making under complex conditions (Kang et al. 2021). ChatGPT was also tested in emergency triage scenarios, comparing its decision‐making capabilities with expert nurses to evaluate its potential as a support tool (Saban and Dubovi 2024).

Long‐Term Care (LTC): In LTC settings, AI supports early identification of patient care needs and decision‐making, particularly for monitoring and prioritizing high‐risk patients (Sadeghi et al. 2024). AI also supports daily care planning, monitoring health status, and reducing caregiver workload (Lukkien et al. 2024).

Remote Monitoring and Patient Safety: AI applications have been studied using remote monitoring for fall prediction, vital signs tracking, and medication management to reduce risks and streamline nurse workflows (Bharadwaj et al. 2021; Yelne et al. 2023).

D Factors influencing the study of AI's contribution to nursing decision‐making.

User Acceptance and Training Needs: Trust in AI is influenced by the interpretability and transparency of the models, as nurses need to understand AI recommendations to integrate them confidently into patient care (Stiglic et al. 2020; Yelne et al. 2023). Preference for AI tools and training on their interpretative capabilities influence acceptance, especially among novice nurses (Mahmoudi and Moradi 2024; Martinez‐Ortigosa et al. 2023).

Human‐AI Collaboration and Over‐Reliance Risks: The risk of over‐reliance on AI, such as alarm fatigue where AI‐enabled devices or CDSS produced excessive or poorly timed alerts—leading nurses to experience cognitive overload and reduced vigilance (Kovach and Pollonini 2022; Yelne et al. 2023). Likewise, depersonalization of care with negative consequences for patient experience is highlighted, emphasizing the importance of maintaining a balance between AI and human judgment (Lukkien et al. 2024; Saban and Dubovi 2024).

Ethical and Privacy Concerns: Privacy concerns, especially with data‐sensitive tools, are significant in determining AI's applicability in nursing, emphasizing the need for secure, ethically designed systems (Sadeghi et al. 2024; Stiglic et al. 2020). AI's integration raises concerns about data privacy, potential algorithm bias, and preserving patient autonomy and dignity (Mahmoudi and Moradi 2024; Martinez‐Ortigosa et al. 2023). The ability to explain AI decisions can foster trust, accountability, and acceptance among healthcare providers and helps ensure ethical standards, supporting informed decision‐making in patient care (Sadeghi et al. 2024).

E Implications for future research.

Need for Empirical Evidence: There is a call for more empirical research into how AI is used in real‐world nursing practices enabling understanding of its practical impact and cultivating its applications (Al Khatib and Ndiaye 2025). Further studies are needed on AI's long‐term implications for nursing practices, especially in specific settings like LTC and emergency care (Martinez‐Ortigosa et al. 2023).

Development of Context‐Specific AI Models: Future research should focus on developing context‐specific AI models that adapt to the dynamic needs of different care settings and address the unique challenges in each environment, such as ICUs and LTCs (Dweekat et al. 2023; Kang et al. 2021). Research should focus on creating models tailored to particular nursing contexts, addressing specific needs and ethical standards in LTC and critical care (Mahmoudi and Moradi 2024; Saban and Dubovi 2024).

Interdisciplinary Collaboration: Collaboration across fields, including ethics, healthcare, and data science, is essential for creating AI systems that are both effective and aligned with healthcare standards (Yelne et al. 2023). Successful AI integration requires interdisciplinary collaboration to ensure ethical design, practical implementation, and user‐centered approaches (Lukkien et al. 2024; Mahmoudi and Moradi 2024).

## 5 Discussion

This scoping review provides important knowledge on AI's contributions to nursing decision‐making, mapping and systematizing the existing evidence.

The findings highlight AI's potential to enhance diagnostic accuracy, streamline workflows, and improve patient outcomes, especially in intensive care and critical healthcare settings. However, interpretability remains a challenge, influencing AI adoption in nursing.

The review encompasses both original and secondary studies. Integrating secondary studies is justified because they offer consolidated insights that are not apparent when examining individual primary studies alone. Across all studies, there is a consistent emphasis on addressing ethical, legal, and technical barriers to ensure the effective integration of AI into nursing practice, ultimately strengthening evidence‐based decision‐making and improving healthcare quality.

### 5.1 The Role of Explainable AI in Enhancing Nursing Decision‐Making

One of the major challenges in adopting AI technologies is ensuring that the decision‐making processes of these models are interpretable and transparent, allowing nurses and other healthcare professionals to clearly understand how AI‐driven decisions are made, mainly when patient safety is a concern (Carvalho et al. 2019).

XAI addresses this need by elucidating the mechanisms behind AI models, ensuring their outputs are both interpretable and reliable (Bharadwaj et al. 2021; Sadeghi et al. 2024). This set of processes uses a diversity of methods to improve interpretability, such as Shapley Additive Explanations (SHAP) and Local Interpretable Model‐agnostic Explanations (LIME). SHAP measures each input feature's contribution to the output of a model and is based on cooperative game theory. In nursing, SHAP helps clarify how particular patient information, including clinical symptoms or test results, affects prognoses for illnesses like sepsis or cardiac arrhythmia. With this specificity, nurses can adjust their clinical interventions according to AI‐generated risk considerations (Carvalho et al. 2019; Lundberg 2017; Sadeghi et al. 2024). This method enhances nurses' clinical decision‐making by revealing how variables like prescription adjustments and vital signs shape the results produced by AI systems. Because LIME is a model‐agnostic method classifier, it may be used in different AI systems, increasing its adaptability in various healthcare settings. It enables nurses to comprehend the rationale behind a machine learning model's prediction (Barr Kumarakulasinghe et al. 2020; Erdeniz et al. 2023).

These advantages highlight XAI's added value to nurses making decisions (Bharadwaj et al. 2021; Esteva et al. 2019; Johnson et al. 2016; Knapič et al. 2021). In addition to aiding nurses in individual clinical decision‐making, explainable AI (XAI) provides broader advantages, including support for early detection and rapid identification of suspected cases. By enabling more accurate and timely diagnoses, XAI contributes to improved patient outcomes, reduces the risk of misdiagnosis, and helps mitigate challenges associated with limited access to advanced diagnostic infrastructure (Pongsuwun et al. 2025).

XAI is also essential in promoting a sense of reliability and confidence in AI systems by improving the interpretability of AI models and allowing nurses to trust these tools without losing their professional judgment (Pongsuwun et al. 2025). According to Kahraman et al. (2025), in a recent study, they state that artificial intelligence can enhance workload management, clinical decision‐making, and patient safety. However, nurses must develop the competence to critically assess and ethically implement these tools. Building trust is a fundamental prerequisite for the effective integration of AI in healthcare practice (Kahraman et al. 2025). Moreover, XAI increases interdisciplinary collaboration by presenting accurate explanations that improve interaction between nurses, physicians, and other healthcare workers (Tonekaboni et al. 2019). This collaborative approach has the potential to reduce diagnostic errors and enhance patient outcomes, particularly in high‐risk conditions where timely intervention and early diagnosis are critical. For instance, researchers in Germany are at the forefront of efforts to enhance the explainability and transparency of AI for healthcare professionals, including nurses. Institutions such as the German Research Center for Artificial Intelligence have conducted studies aimed at developing XAI models specifically designed for clinical applications. These studies highlight the critical role of interpretable AI in clinical settings, where nurses must be able to understand and evaluate AI‐generated recommendations for patient care (Sadeghi et al. 2024).

Likewise, AI models for diagnosis support (e.g., early detection of sepsis) are being tested in collaboration with nurses to ensure usability and trust (Pongsuwun et al. 2025). A study from the Charité—Universitätsmedizin, Berlin focuses on developing XAI models to assist nurses in monitoring patients in different fields of medicine, explaining and highlighting AI predictions (Schapranow et al. 2023). These studies help create realistic and personalized scenarios, in which AI can significantly strengthen through simulation‐based education and thus support nurses' learning needs. As Saban and Dubovi (2025) state, these experiences play a pivotal role in guiding nurses toward the provision of a structured reasoning and thus, enhancing confidence and clinical (Mahmoudi and Moradi 2024; Martinez‐Ortigosa et al. 2023) In alignment with this perspective, Kahraman et al. (2025) argue that continuous training initiatives are essential to close these existing gaps, and that nursing curricula must incorporate AI literacy alongside practical applications (Kahraman et al. 2025).

CDSS, developed by AI models, are broadly used to provide crucial support to nurses in high‐risk circumstances, such as impending death situations, where CDSS can assist nurses in achieving efficient therapies, anticipating patient clinical decline, and improving treatment. Based on patient data, these systems offer real‐time recommendations, providing a safety net for nurses and enabling early therapies that can prevent adverse outcomes like hospital‐acquired diseases or mortality. AI models skilled in evaluating large datasets and identifying subtle patterns in medical imaging and biometrics improve nursing interventions by providing accurate insights that may not be immediately evident (Jalali et al. 2016). Therefore, XAI promotes transparency in AI models by rendering their decisions comprehensible. In contrast, Clinical Decision Support Systems (CDSS) provide guidance and recommendations to enhance patient care and may incorporate XAI techniques to strengthen interpretability.

Consecutively, Saban and Dubovi (2024), focusing on ChatGPT as an instrument with potential application in analyzing the performance of healthcare in clinical decision‐making, conclude that ChatGPT exhibited indecisiveness in initial assessments and tends to be over‐triggered by recommending unnecessary diagnostic tests. On the other hand, when a reassessment was necessary, ChatGPT's responses were often inaccurate or inappropriate when modifying decisions. Although ChatGPT responded more quickly than nurses, it did not translate into safer or more appropriate clinical reasoning, highlighting that fast outputs over judgment do not compensate for effective decision‐making. However, it can significantly improve elements of the decision by transforming words into proxies for action, although ChatGPT can't consistently match human nurses' clinical decision‐making capabilities, particularly in urgent, complex scenarios. Despite its utility as a support tool, its current form of clinical decision‐making has significant limitations and risks that must be carefully considered (Saban and Dubovi 2024).

### 5.2 Methods Used to Study AI's Contribution to Nursing Decision‐Making

The review reveals various methodologies employed to study AI's contribution to nursing decision‐making, focusing on model development and validation studies. A significant portion of the research is centered on developing AI models, such as neural networks and recurrent neural networks (RNNs), that are trained and tested using retrospective clinical data from large‐scale databases like Multiparameter Intelligent Monitoring in Intensive Care (MIMIC‐III) and Electronic Intensive Care Unit (eICU) (Dweekat et al. 2023; Yelne et al. 2023). These databases cover a broad patient information, such as vital signs, laboratory results, medication history, and clinical outcomes, supporting a wide range of studies in epidemiology, decision‐rule development, and electronic tool design, essential for predicting key patient outcomes.

Studies frequently use performance metrics such as the Area Under the Curve (AUC), accuracy, and F‐scores to evaluate the models' performance in predicting patient outcomes, including in‐hospital mortality, disease progression, and risk stratification (Jalali et al. 2016; Johnson et al. 2016). These metrics are critical in evaluating the practical utility of AI models in clinical practice, as they provide an indication of the model's predictive capacity and potential integration into existing workflows. For example, Shickel et al. (2018) demonstrated how Long Short‐Term Memory (LSTM) networks applied to MIMIC‐III data achieved high AUC scores in predicting in‐hospital mortality, indicating the potential of AI to enhance decision‐making in critical care settings (Shickel et al. 2018). Neural networks, particularly RNNs and their variants like (e.g., LSTM) networks, are frequently used due to their ability to capture temporal dependencies in clinical data, making them well‐suited for handling real time‐series data such as vital signs and laboratory results (Lipton et al. 2015). These models allow real‐time decision support, a crucial aspect in ICU care environments (Jalali et al. 2016; Kang et al. 2021; Kovach and Pollonini 2022; Popernack 2006). Thus, these performance models act like a built‐in guide, highlighting which parts of a patient's data the model is focusing on when making a prediction. Nevertheless, the key challenge in AI adoption, within nursing practice, is the interpretability of the models. Studies reveal the need to enhance AI models interpretability, so that nurses may understand the need action (Kovach and Pollonini 2022). As these models prioritize monitoring and interventions around certain input or time points variables, that are aligned with clinical reasoning and significant clinical outcomes, they grant nurses a sense of confidence and transparency (Choi et al. 2019; Jalali et al. 2016; Kang et al. 2021; Stiglic et al. 2020). As some authors state, transparency is a critical factor in attracting attention to models, primarily because it builds trust, facilitates scrutiny, and allows nurses to understand AI tools value, limitations and adoption (Sadeghi et al. 2024).

For example, currently, some models predict sepsis, so these models have mechanisms that could highlight specific vital sign analyses (e.g., increasing heart rate or decreasing blood pressure) and critical time points (e.g., within 24 h before onset) that significantly contribute to the prediction (Jalali et al. 2016). This increases the model's interpretability, as it empowers nurses to intervene more efficiently by recognizing which variables require closer monitoring, but also how this data influences outcomes.

### 5.3 Contexts in Which AI's Contribution to Nursing Decision‐Making Has Been Studied

Research using AI in nursing decision‐making across various clinical contexts has focused on acute and ICUs settings. Studies repeatedly emphasize how AI supports nurses in making critical, time‐sensitive decisions, such as predicting a patient's death in an ICU or monitoring indications of sepsis or acute respiratory failure. AI models are included in CDSS in ICUs to provide nurses with real‐time patient data and practical insights, like early warning indicators of worsening clinical conditions. Studies focusing on ICUs and acute care settings indicate that these environments are particularly suitable for the implementation of AI innovations (Jalali et al. 2016; Kang et al. 2021; Kovach and Pollonini 2022; Popernack 2006).

Similarly, AI has also been studied in chronic disease management, where models help nurses monitor and predict outcomes for patients with long‐term conditions, such as diabetes or heart failure. AI models have also been applied to long‐term care, where nurses can prevent pressure ulcers, guide personalized care strategies, enhance shared decision‐making, and reduce caregiver workload (Lukkien et al. 2024). Nevertheless, the transfer of AI models to less acute settings (Martinez‐Ortigosa et al. 2023), such as chronic care or outpatient services, requires further investigation. This need is illustrated by studies conducted in the pressure ulcer field (Dweekat et al. 2023) and in long‐term care environments (Lukkien et al. 2024). Future research should fill this gap by developing AI systems that can be adapted to a broader range of nursing contexts, thus increasing the use of AI in nursing decision‐making across healthcare systems (Kahraman et al. 2025).

#### 5.3.1 Factors Influencing the Study of AI's Contribution to Nursing Decision‐Making

Several factors have been identified as influencing the study and application of AI in nursing decision‐making. A key factor is the interpretability of AI models, already mentioned in this review where nurses must understand the basis behind AI projections in order to trust and effectively use these tools in practice (Pongsuwun et al. 2025). Research emphasizes that enhancing interpretability increases the likelihood of AI systems being adopted in clinical settings, as healthcare professionals require transparency to make informed decisions (Choi et al. 2019; Jalali et al. 2016; Kang et al. 2021; Stiglic et al. 2020).

Another significant element is the incorporation of AI tools into existing clinical workflows. AI systems that integrate seamlessly into nursing workflows, without introducing additional difficulties, are more likely to be adopted. This is largely due to the inherent complexity of these workflows and the resistance to change when new technologies disrupt established practices (Saif‐Ur‐Rahman et al. 2023). An additional barrier to AI implementation arises from the monitoring practices of nurses' work (e.g., alarm systems), particularly when these contribute to exhaustion or workflow disruption, regulatory and management failures or data quality, which may ultimately reflect a flawed realization of AI integration (Bharadwaj et al. 2021; Popernack 2006; Stiglic et al. 2020). Conversely, certain factors may facilitate the implementation of these systems, such as AI solutions with intuitive interfaces that employ visual signals (e.g., color‐coded alerts) and co‐production with nurses to convey critical information, which tend to be more readily accepted. By alleviating cognitive overload and fitting seamlessly into nurses' routines, they act as catalysts for nursing practice transformation (Kovach and Pollonini 2022; Stiglic et al. 2020).

One of the main issues in the literature is the generalizability of AI models across different hospitals. Findings of this review show that AI models should be validated across diverse populations and healthcare settings (Lukkien et al. 2024; Stiglic et al. 2020; Yelne et al. 2023). This aligns with the findings of Esteva et al. (2019), who points out that although AI models are highly accurate in controlled environments, their effectiveness is often reduced in new or untested clinical contexts (Esteva et al. 2019). Moreover, ethical concerns related to data privacy and bias in AI models have been highlighted as significant factors in recent studies (Bharadwaj et al. 2021; Sadeghi et al. 2024; Stiglic et al. 2020; Yelne et al. 2023). Ethical concerns like transparency, equality, and data privacy demand permanent attention (Yelne et al. 2023). Likewise, a recent study adds that, although most of the existing instruments measure general AI literacy (e.g., technical competence, self‐efficacy, or general knowledge of AI systems), most fail to capture ethical dimensions specific to nursing (El‐Sayed et al. 2025). In parallel, research and international public health agencies tasked with coordinating responses to global health crises highlight the need to design AI systems that not only deliver outstanding performance but also embed robust ethical standards, (Markets and Markets 2024), ensuring equality and preventing inadvertent biases that could affect patient care (Jeyaraman et al. 2023; Yelne et al. 2023). Requirements that need a collaborative interdisciplinary approach in the development of these AI systems involving AI developers, nurses, and ethicists. This review supports the need for more ethical oversight in the development of AI systems, particularly when they are being integrated into critical decision‐making processes like nursing practice.

Considering the foregoing, AI contributes to nursing by enhancing clinical decision‐making, anticipating risks, and reducing practice variability, whereas simultaneously promoting patient safety through early detection and timely interventions. Beyond direct patient care, AI holds the potential to optimize resources, alleviate workload, and improve organizational efficiency. In nursing education, it supports simulation‐based learning and the development of clinical reasoning, underscoring the need for both digital and ethical literacy (Pongsuwun et al. 2025). Likewise, from a scientific‐methodological standpoint, AI integration reinforces the importance of standardization, the establishment of nursing‐specific metrics, and the implementation of studies that critically evaluate its real impact on practice.

Regarding study's limitations, the application of AI in nursing care is still an emerging field with limited evidence, as available studies show significant heterogeneity in AI types and settings. As a result, generalizations of the findings should be approached with caution. Although terms like “artificial intelligence,” “technology‐enabled learning,” “artificial neural networks,” and “machine learning” were used to capture AI methods, the heterogeneity of studies and languages may have constrained access to pertinent literature. Despite the limitations, this review significantly contributes to our current understanding of AI‐based systems in nursing practice. Although positive outcomes were demonstrated for most AI applications, it's clear that more studies are needed to address these systems' limitations and better understand the needs of healthcare professionals in their development. This underscores the significant potential for further advancement, particularly in ensuring professional autonomy and improving access to health information sources. These improvements would optimize the use of AI in multitasking, considering the many variables that impact patients, environments, clinical practices, and medical services. Further research is needed to explore the application of AI systems in real‐world clinical nursing practice, with the aim of enabling more informed and deliberate decision‐making. Additionally, more investigation is required to assess how AI support systems enhancing patient safety and assist nurses in decision‐making within specific clinical settings.

### 5.4 Implications for Future Research

This review conclusively points out the gaps that further studies can explore. One way is to conduct more clinical trials and real‐world validations of these AI models in nursing (Carvalho et al. 2019; Erdeniz et al. 2023; Saif‐Ur‐Rahman et al. 2023; Tonekaboni et al. 2019), particularly in diverse clinical settings, and not restricted solely to ICUs settings. In addition to the ethical challenges posed by AI bias and data privacy, further considerations are directed toward guaranteeing the trustworthiness and transparency of AI‐driven decision‐making (El‐Sayed et al. 2025). Future research should also explore hybrid AI models that mix interpretable frameworks with the predictive power of more complex models so that nurses can trust and effectively use AI tools in their decision‐making (Kang et al. 2021; Popernack 2006; Sadeghi et al. 2024).

Moreover, this review emphasizes the importance of producing AI systems with technical rigor and clinical applicability through interdisciplinary collaboration by several healthcare professionals, such as nurses, physicians, AI developers and ethicists. Collaboration with nurses, providing confidence in AI instruments, is paramount for developing systems that are supportive to nursing practices dynamics, and thus its acceptance and potential recognition (Dal Mas et al. 2023; Stiglic et al. 2020; Yelne et al. 2023).

## 6 Conclusions

This review maps the growing evidence of AI's role in nursing decision‐making, emphasizing its contribution through explainable models in improving diagnostic accuracy, streamlining workflows, and enhancing patient safety. However, AI integration requires careful balancing with ethical considerations and human oversight to safeguard critical thinking, empathy, and decision‐making in nursing. The role of AI is to complement, not supplant these critical skills, ensuring the delivery of safe, personalized, and high‐quality care.

Although AI holds transformative potential, ethical, legal, social, and technical challenges must be addressed. Accuracy, informed consent, and data privacy necessitate robust legislation and public engagement. Regulating liability and ensuring equitable access to AI‐driven healthcare solutions are essential for building trust and inclusivity.

Technical challenges, including data scarcity, model robustness, and workflow integration, require further research, particularly in real‐time inference and explainability. Addressing these challenges will enable AI to support better outcomes in healthcare. This review highlights AI's potential to revolutionize nursing decision‐making, particularly in high‐complex settings, and underpins the need for further research to ensure its ethical and effective integration into practice.

## Author Contributions

Conceptualization: Filipe Fernandes; Lucy Shinners; Mauro Mota; Paulo Santos and Luís Sá. Methodology: Filipe Fernandes; Lucy Shinners; Mauro Mota; Paulo Santos and Luís SÁ. Formal analysis and investigation: Filipe Fernandes; Lucy Shinners; Mauro Mota; Paulo Santos and Luís Sá. Writing – original draft: Filipe Fernandes; Lucy Shinners; Mauro Mota; Paulo Santos and Luís Sá. Writing – review and editing: Filipe Fernandes; Lucy Shinners; Mauro Mota; Paulo Santos and Luís Sá. Funding acquisition: This research did not receive any specific grant from funding agencies (public, commercial, or not‐for‐profit sectors). Supervision and validation: Luís Sá, Though, all authors whose names appear on the submission agree to be accountable for all aspects of the study in ensuring that questions related to the accuracy or integrity of any part of the it, were appropriately outlined.

## Funding

The authors have nothing to report.

## Disclosure

Relevance for clinical practice: The application of AI to real‐life clinical nursing practice benefits and supports more conscious clinical decisions. By identifying patterns and providing evidence‐based recommendations, these AI‐driven systems can enable timely interventions and thus enhance efficiency, accuracy and significantly contribute to earlier detection and targeted interventions, with the potential to improve outcomes. Nevertheless, AI applications need comprehensive ethical frameworks tailored to nursing practice. This scoping review aims to explore this balance.

## Ethics Statement

The authors have nothing to report.

## Conflicts of Interest

The authors declare no conflicts of interest.

## Supporting information


**Data S1:** NHS_70308_sup_0001_Data S1.docx.

## Data Availability

Data sharing not applicable to this article as no datasets were generated or analysed during the current study.
