# Supplementary material for: Contributions of Artificial Intelligence to Decision Making in Nursing: A Scoping Review
Source: Nurs Health Sci. 2026 Feb 18;28(1):e70308. doi: 10.1111/nhs.70308 (PMC12917350; doi:10.1111/nhs.70308)
Supplement: Supplementary file 1 — Data S1: NHS_70308_sup_0001_Data S1.docx. [file NHS-28-e70308-s001.docx]

**Appendix 1**

The search strategies were developed using subject headings (MeSH) and free-text terms for each database.

**MEDLINE via PubMed**

| Search | Query |
| --- | --- |
| #1 | ((((((((((((((((((Artificial Intelligence[MeSH Terms]) OR (Computer Science[MeSH Terms])) OR (Logic programing[Title/Abstract])) OR (Entropy[MeSH Terms])) OR (Technology Enable Learning[Title/Abstract])) OR (Social Computing[Title/Abstract])) OR (Artificial Neural Networks[Title/Abstract])) OR (Computational Sustainability[Title/Abstract])) OR (Knowledge Representation[Title/Abstract])) OR (Clinical Reasoning[MeSH Terms])) OR (Big data[MeSH Terms])) OR (Digital transformation[Title/Abstract])) OR (Natural language processing[MeSH Terms])) OR (Data analytics[MeSH Terms])) OR (Machine learning[MeSH Terms])) OR (Deep learning[MeSH Terms])) OR (Medical Records Systems, Computerized[MeSH Terms])) OR (Natural language processing[MeSH Terms])) OR (Emerging technologies[Title/Abstract]) |
| #2 | (((((((Clinical Decision-Making[MeSH Terms]) OR (Decision-making[Title/Abstract])) OR (Clinical decision[Title/Abstract])) OR (Decision Support Systems, Clinical[MeSH Terms])) OR (Nursing Diagnosis[MeSH Terms])) OR (Nursing[MeSH Terms])) OR (Nursing[Title/Abstract])) OR (Nurs*[Title/Abstract]) |
| #3 | ((((((Intensive Care Units[MeSH Terms]) OR (Critical Care Nursing[MeSH Terms])) OR (Critical patient[Title/Abstract])) OR (Critical Care Nursing[Title/Abstract])) OR (Intensive Care Units[Title/Abstract])) OR (Emergency Medical Services[MeSH Terms])) OR (Emergency Medical Services[Title/Abstract]) |
| #4 | #1 AND #2 AND #3 |

**CINAHL (EBSCO)**

| **Search** | **Query** |
| --- | --- |
| S1 | (AB Artificial Intelligence OR AB Computer Science OR AB Logic programing OR AB Entropy OR AB Technology Enable Learning OR AB Social Computing OR AB Artificial Neural Networks OR AB Computational Sustainability OR AB Knowledge Representation OR AB Clinical Reasoning OR AB Big data OR AB Digital transformation OR AB Natural language processing OR AB Data analytics OR AB Machine learning OR AB Deep learning OR AB Computerized Medical Records Systems OR AB Natural language processing OR AB Emerging technologies) |
| S2 | (AB Clinical Decision-Making OR AB Decision-making OR AB Clinical decision OR AB Clinical Decision Support Systems OR AB Nursing Diagnosis OR AB Nursing OR AB Nurs*) |
| S3 | (TX Intensive Care Units OR TX Critical Care Nursing OR TX Critical patient OR TX Critical Care Nursing OR TX Intensive Care Units OR TX Emergency Medical Services OR TX Emergency Medical Services) |
| S4 | (S1 AND S2 AND S3) |

**Scopus**

| **Search** | **Query** |
| --- | --- |
| #1 | (TITLE-ABS-KEY (Artificial Intelligence OR Computer Science OR Technology Enable Learning OR Logic programing OR Technology Enable Learning OR Entropy OR Social Computing OR Artificial Neural Networks OR Computational Sustainability OR Knowledge Representation OR Clinical Reasoning OR Big data OR Digital transformation OR Natural language processing OR Data analytics OR Machine learning OR Deep learning OR Computerized Medical Records Systems OR Natural language processing OR Emerging technologies)) |
| #2 | (TITLE-ABS-KEY (Clinical Decision-Making OR Decision-making OR Clinical decision OR Clinical Decision Support Systems OR Nursing Diagnosis OR Nursing)) |
| #3 | (TITLE-ABS-KEY (Intensive Care Units OR Critical Care Nursing OR Critical patient OR Critical Care Nursing OR Intensive Care Units OR Emergency Medical Services OR Emergency Medical Services) |
| #4 | (TITLE-ABS-KEY(Artificial Intelligence OR Computer Science OR Technology Enable Learning OR Logic programing OR Technology Enable Learning OR Entropy OR Social Computing OR Artificial Neural Networks OR Computational Sustainability OR Knowledge Representation OR Clinical Reasoning OR Big data OR Digital transformation OR Natural language processing OR Data analytics OR Machine learning OR Deep learning OR Computerized Medical Records Systems OR Natural language processing OR Emerging technologies) AND (TITLE-ABS-KEY(Clinical Decision-Making OR Decision-making OR Clinical decision OR Clinical Decision Support Systems OR Nursing Diagnosis OR Nursing) AND (TITLE-ABS-KEY(Intensive Care Units OR Critical Care Nursing OR Critical patient OR Critical Care Nursing OR Intensive Care Units OR Emergency Medical Services OR Emergency Medical Services)) |

**APA PsycINFO (EBSCO)**

| **Search** | **Query** |
| --- | --- |
| S1 | AB Artificial Intelligence OR AB Computer Science OR AB Logic programing OR AB Entropy OR AB Technology Enable Learning OR AB Social Computing OR AB Artificial Neural Networks OR AB Computational Sustainability OR AB Knowledge Representation OR AB Clinical Reasoning OR AB Big data OR AB Digital transformation OR AB Natural language processing OR AB Data analytics OR AB Machine learning OR AB Deep learning OR AB Computerized Medical Records Systems OR AB Natural language processing OR AB Emerging technologies |
| S2 | AB Clinical Decision-Making OR AB Decision-making OR AB Clinical decision OR AB Clinical Decision Support Systems OR AB Nursing Diagnosis OR AB Nursing |
| S3 | S1 AND S2 |

**LILACS**

| **Search** |
| --- |
| (Technology Enable Learning OR Artificial Intelligence OR Computer Science OR Knowledge Representation OR Clinical Reasoning OR Machine learning) [Palavras] and Clinical Decision-Making OR Decision-making OR Clinical decision OR Clinical Decision Support Systems OR Nursing Diagnosis OR Nursing [Palavras] |

**JBI Evidence Synthesis:** Searched on September 28, 2023

| **Search** |
| --- |
| *Artificial Intelligence; Technology Enable Learning* |

**Cochrane Library**

| **Search** | **Query** |
| --- | --- |
| #1 | (Logic programing OR Technology Enable Learning OR Social Computing OR Artificial Neural Networks OR Computational Sustainability OR Knowledge Representation OR Digital transformation OR Emerging technologies):ti,ab,kw |
| #2 | MeSH descriptor: [Artificial Intelligence] explode all trees |
| #3 | MeSH descriptor: [Computer Science] explode all trees |
| #4 | MeSH descriptor: [Entropy] explode all trees |
| #5 | MeSH descriptor: [Clinical Reasoning] explode all trees |
| #6 | MeSH descriptor: [Big data] explode all trees |
| #7 | MeSH descriptor: [Natural language processing] explode all trees |
| #8 | MeSH descriptor: [Data analytics] explode all trees |
| #9 | MeSH descriptor: [Machine learning] explode all trees |
| #10 | MeSH descriptor: [Deep learning] explode all trees |
| #11 | MeSH descriptor: [Medical Records Systems, Computerized] explode all trees |
| #12 | MeSH descriptor: [Natural language processing] explode all trees |
| #13 | (Decision-making OR Clinical decision OR Nursing):ti,ab,kw |
| #14 | MeSH descriptor: [Clinical Decision-Making] explode all trees |
| #15 | MeSH descriptor: [Decision Support Systems, Clinical] explode all trees |
| #16 | MeSH descriptor: [Nursing Diagnosis] explode all trees |
| #17 | MeSH descriptor: [Nursing] explode all trees |
| #18 | (#1 OR #2 OR #3 OR #4 OR #5 OR #6 OR #7 OR #8 OR #9 OR #10 OR #11 OR #12) AND (#3 OR #14 OR #15 OR #16 OR #17) |

**RCAAP – Portuguese Open Access Scientific Repository**

| **Search** | **Query** |
| --- | --- |
| Full-text: Artificial Intelligence OR Technology Enable Learning | |

**Theses & Dissertations Catalogue - CAPES**

| **Search** |
| --- |
| Artificial Intelligence OR Technology Enable Learning |
| Language limits (English, French, Spanish and Portuguese) |
